# Assessing public–private procurement practices for medical commodities in Dar Es Salaam: a situation analysis

**DOI:** 10.1186/s12913-022-08923-1

**Published:** 2022-12-14

**Authors:** Romuald Mbwasi, Denis Mbepera, William Mfuko, Jason Makanzo, Martha Kikwale, Robert Canavan, Manfred Stoermer, Karin Wiedenmayer

**Affiliations:** 1 St. John’s University of Tanzania, Dodoma, Tanzania; 2 Ministry of Health, Dodoma, Tanzania; 3 Senior freelance pharmaceutical consultant, Dar es Salaam, Tanzania; 4 Regional Administrative Secretary’s Office, Dar es Salaam, Tanzania; 5 Swiss Tropical and Public Health Institute, P.O. Box, CH-4123 Allschwil, Switzerland; 6 University of Basel, P.O. Box, CH-4003 Basel, Switzerland

**Keywords:** Private supplier, Prime vendor system, Jazia, Public–private partnership, Tanzania, Health system, Pharmaceutical procurement, Medicines management

## Abstract

**Background:**

In Tanzania, the Medical Stores Department is the principal pharmaceutical provider to public health facilities throughout the country. However, growing demand from health facilities has proved difficult to satisfy and stock-outs at health facilities are frequent. The aim of the current study was to conduct a situation analysis of the procedures and practices of procuring medicines and medical supplies from private suppliers in the Dar es Salaam region when those commodities are unavailable at the Medical Stores Department.

**Methods:**

A mixed-method approach including qualitative and quantitative methods was applied to understand procurement procedures and practices and private suppliers’ performance at district level. Qualitative interviews with suppliers and district authorities, and a review of inventory documents at store level was conducted between February and March 2018. The quantitative approach included a review and analyses of relevant procurement documents from the 2016/2017 financial year to explore the funds used to procure health commodities from the private sector. The ten most frequently mandated private suppliers were assessed in more detail focusing on cost, quality and availability of medicines and lead times and delivery.

**Results:**

A lack of consistency and written guidelines for procuring medicines and medical supplies from the private sector was observed. The procurement process was bureaucratic and lengthy requiring multiple steps between health facilities, suppliers and district authorities. A significant number of people were involved requiring a minimum of 13 signatures and 16 steps from order preparation to approval.

Only 17 of 77 prequalified private suppliers received orders from public health facilities. The criteria for choosing which supplier to use were unclear. Completed orders amounted to USD 663,491. The bureaucratic process drove councils and healthcare facilities towards alternative ways to procure health commodities when Medical Stores Department stock-outs occurred.

**Conclusion:**

The procurement procedure outside the Medical Stores Department is inefficient and cumbersome, often circumventing government regulations. General lack of accountability renders the process susceptible to leakage of funds and medicines. Increasing the transparency and efficiency of procurement procedures from the private sector with a prime vendor system would help to better manage Medical Stores Department stock-outs and help improve health care services overall.

## Background

Strengthening a national health care system is a complex, country-specific process that is shaped by national demographics, history and culture, the epidemiology and the resources available [1]. Tanzania’s overall health policy objective is to strengthen the national healthcare system and to, eventually, provide universal health coverage (UHC) in accordance with the Alma-Ata declaration [2, 3]. The intention to steer progress towards its Development Vision 2025 and the United Nations Sustainable Development Goal 3 (“Good health and wellbeing”) is ambitious. This is especially so considering the complex history of Tanzania’s healthcare system, which has been overshadowed by critical shortages of skilled healthcare workers and inadequate financial and physical resources [4–6]. The general perception of public healthcare services, by the population particularly in rural peripheral facilities, is one of poor quality of care, shortages of medicines and medical supplies and corruption [2, 7]. In Tanzania, the Medical Stores Department is the principal provider of pharmaceuticals and the sole public organization responsible for procurement, storage and distribution of health commodities to all public health facilities throughout the country. All public providers and approved faith-based organizations send orders to the Medical Stores Department that then delivers the supplies to the facility at all levels. It is a financially independent and non-profit government department under the auspices of the Ministry of Health (MoH) [8]. The growing demands on the Medical Stores Department to fully fill orders for all health facilities in urban, rural and remote areas has proved difficult to satisfy. A strategic review exposed a systemic failure within the supply chain at all levels that contributed to stock-outs at health service delivery points [9–11]. A comprehensive baseline study in 2011 in the Dodoma region revealed that only 53% of the essential 24 tracer medicines were available at public health facilities and 47% were out of stock. In addition, only 59% of medicines ordered from the Medical Stores Department were actually delivered [10]. Under the umbrella of the Public Procurement Act 2011, councils and public health facilities can procure health commodities from prequalified private suppliers when stock-outs occur at the Medical Stores Department using complementary funds [12]. These complementary funds are derived from the Community Health Fund, the National Health Insurance Fund, Health Basket Funds and user fees and they can be applied for at facility level. Purchase from private suppliers can only be initiated once The Medical Stores Department provides an out-of-stock notice. However, the procurement process outside the Medical Stores Department is lengthy, bureaucratic and costly, not least due to the small quantities of high-priced medicines purchased from private suppliers within or outside of a given region. These suppliers − also referred to as vendors − are generally wholesalers/distributors. In addition, the quality of medicines from some private suppliers remains questionable, the procurement process ambiguous and there is a general lack of accountability rendering the process susceptible to corruption and leakage of funds and medicines [7, 10].

The Health Promotion and System Strengthening (HPSS) project was introduced to Tanzania in 2011 to support the government with their effort to comprehensively reform health financing, medicines management, health promotion and technology management within the health sector [13]. The HPSS project piloted a public–private procurement scheme – the Jazia Prime Vendor System (PVS) – in the Dodoma region in 2014 to complement the Medical Stores Department supply chain when stock-outs occurred [10]. The public procurement process from private medical suppliers is defined in the following documents: The Public Procurement Act 2011 and its Regulations (2013) and 2016 Amendments, the Local Government Authority Procurement Regulations and Guidelines for the Management of Funds. The implementation of the Jazia PVS followed a sequence of 12 steps starting with a baseline study quantifying the medicine needs, analysing the current private procurement practices and the financial management of the complementary funds and the procedures to obtain those funds [10]. The Jazia PVS pilot intervention in the region of Dodoma saw the availability of essential medicines increase from 69% in 2014 to 94% in 2018. In 2016, the scheme was successfully extended to the regions of Morogoro and Shinyanga [14]. Following the success of the pilot intervention, the Tanzanian government then requested for the Jazia programme to be rolled-out nationwide [10].

The aim of the current study was to conduct a situation analysis, before implementation of the complementary Jazia PVS, assessing the existing procedures and practices of procuring medicines and medical supplies from private suppliers in the Dar es Salaam region when those commodities are not available at the Medical Stores Department.

## Methods

### Study design and setting

A baseline study was conducted, between the 14th February and the 8th March 2018 in the Dar es Salaam region, Tanzania. Five districts were included, i.e., Ilala, Temeke, Ubungo, Kigamboni and Kinondoni. Prior to the study, suppliers serving the district were identified in each district. The subsequent district study employed both a qualitative and quantitative approach to understand procurement procedures and practices and suppliers’ performance at council level.

### Qualitative and quantitative assessments

The qualitative approach followed a narrative structure using face-to-face interviews with suppliers based on semi-structured questionnaires. Moreover, a documentary review was conducted of the stores focussing on purchase orders, fulfilment by the suppliers, invoices including unit prices, lead time, comparing the suppliers given the orders with the list of prequalified suppliers for the councils. In addition, storage conditions were inspected. The interviews with district authorities focused on open discussions regarding procedures and procurement practices. During the course of the study, the 10 most frequently mandated prequalified suppliers that supplied in the review period (*N* = 50; 17 qualified and 33 non-qualified suppliers) were visited and interviewed. This was done with the purpose of ascertaining their business status and to evaluate their daily operations. The interview teams included council pharmacists or procurement officers for any council other than their own. Interviewees were purposely selected from public staff working in the respective councils and from those suppliers with the highest frequency of supply.

The quantitative approach included a review and analyses of orders placed to private suppliers in each district between July 2016 and June 2017 and a review and analyses of local purchase orders (LPOs), invoices and delivery notes to explore the total funds districts used to procure essential health commodities from the private sector. The performance of a sample of the identified suppliers was then assessed focusing on cost, quality and availability of medicines, lead times, delivery and long-term viability. A number of tools were developed for the study including a table documenting all private suppliers sourced to procure medicines and medical supplies, a standardized interview form to document purchasing procedures and practices and whether they accorded with national procurement guidelines, and a semi-structured questionnaire to assess private suppliers. The analysis of the public procurement process from private medical suppliers in Dar es Salaam was based on the Public Procurement Act 2011 and its Regulations (2013) with 2016 Amendments and the Local Government Authority Procurement Regulations and Guidelines for the Management of Funds. In addition, statements of accounts and lists of prequalified suppliers providing a delivery service of health commodities when health facilities are unable to obtain the same at the Medical Stores Department, were included in the analysis.

### Data collection and analysis

Five teams of data collectors, comprised of pharmaceutical and procurement staff from the five districts, were formed. To avoid any bias, teams would not collect data in their own district. The selected teams were trained for two days on data collection, which included field-testing of the tools. Quantitative data were entered immediately into Excel, while qualitative data were recorded and, the same day, reviewed and entered into Excel and/or Word. Qualitative data were coded manually based on the specific objectives of this work and themes were developed and narrated. Data analysis was carried out using Microsoft Excel, SPSS and PSPP. The findings were disseminated at a stakeholder meeting that included the study participants − most of them government workers. They were therefore invited to this dissemination process, followed by an open discussion. There was no objection against the data collected, which confirmed the validity of the data recorded.

### Ethics

Permission for the data acquisition was approved by Regional Secretariat Office, the highest authority in the region. Interviewees were provided with information explaining the rationale for the study, their rights, confidentiality and who will have access to information provided. Informed consent was obtained verbally as described in the approved study protocol.

## Results

### Procurement procedures and practices

**Fig. 1:**
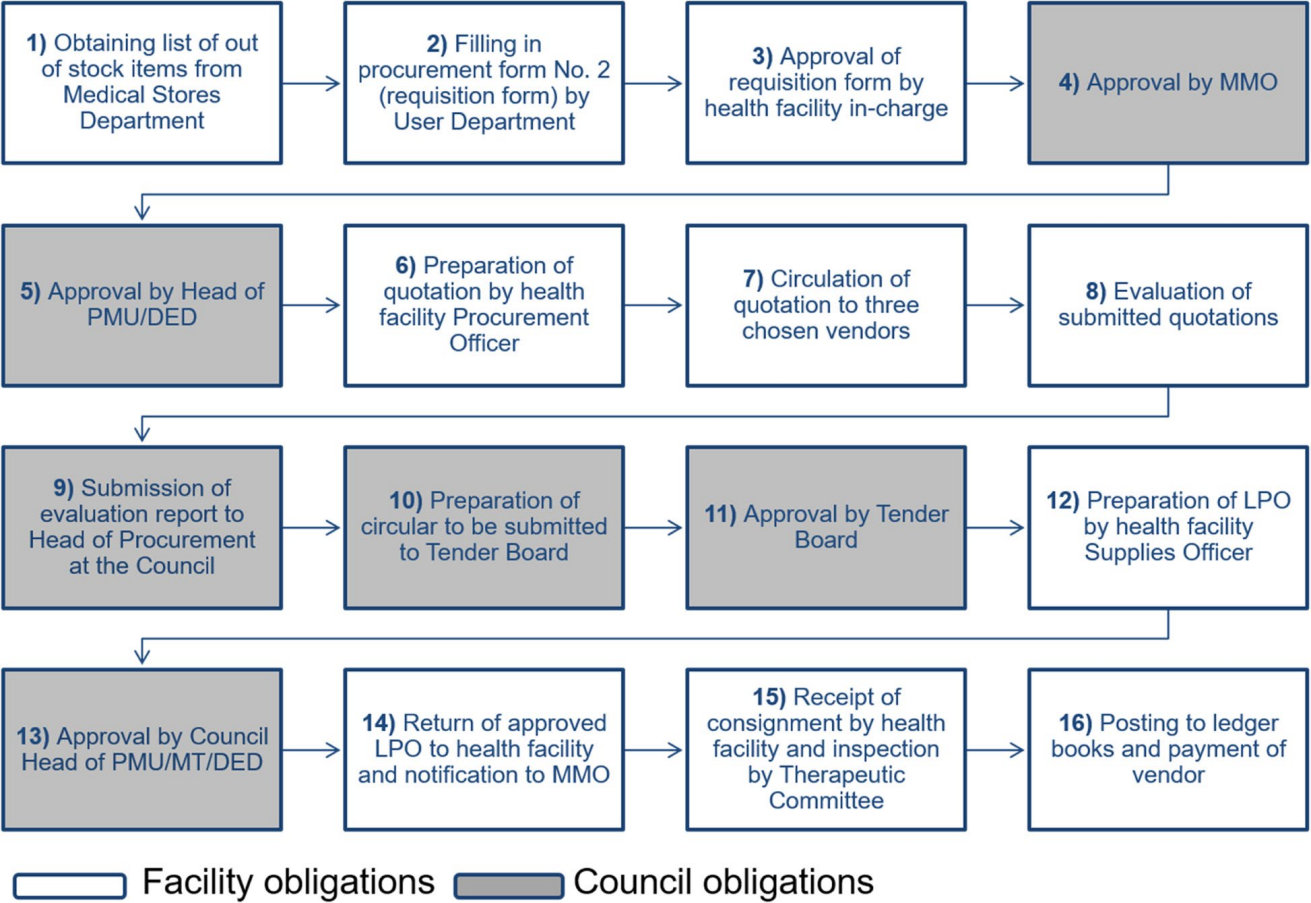
Procurement steps: DED, District Executive Director; LPO, Local purchase order; MMO-Municipal Medical Officer; MT, Municipal Treasurer; PMU, Procurement Management Unit

With regards to the Public Procurement Act 2011 and its Regulations (2013) with 2016 Amendments and the Local Government Authority Procurement Regulations and Guidelines for the Management of Funds, adherence to the legislation, however, was not consistent. None of the districts had written guidelines or SOPs to describe and simplify the necessary steps for procuring medicines from the private sector. The procurement process was bureaucratic and lengthy requiring multiple steps between health facilities, suppliers and district authorities. In addition, a significant number of people were involved in the process requiring a minimum of 13 signatures from order preparation to authorisation and finally to approval. In total, 16 steps were identified for the procurement of health commodities both at district and health facility level. Figure 1 illustrates each step and the approval required by councils and health facilities for procuring commodities for public health facilities outside the Medical Stores Department delivery schedules in the Dar es Salaam region.

Government procurement regulations require that all purchases outside the government system should have the following documentation: approved quotation from a transparently selected supplier, issued local purchase order (LPO) from the facility to the supplier for every order made, an invoice accompanying the goods supplied and finally a cheque or bank transfer for payment authorized by the facility. Each purchase must have a new LPO established, that is an LPO cannot be used repeatedly.

However, councils and healthcare facilities would resort to alternative means to avoid lengthy, bureaucratic and cumbersome procedures for procuring health commodities when MSD stock-outs occurred. Although Tanzanian financial regulations discourage the use of public money in the form of petty cash for routine purchase of healthcare supplies, the study revealed that petty cash was used on a routine basis. An analysis of purchases in one healthcare facility visited revealed 116 complementary purchases; eight were paid for by cheque and for 108, cash was exchanged. Other mechanisms for circumventing the government prescribed procurement process included the utilization of the same LPO for several procurement actions (up to one year), thereby avoiding carry-over balances in district accounts. To ensure the latter, districts would deposit all their allocated Health Basket Funds for health commodities to the Medical Stores Department, regardless of the completeness of supply delivery. This was done to avoid raising audit queries, in a given review period, that may lead to cutting of funds in the subsequent period. Other healthcare facilities simply did not procure any commodities from private suppliers despite stock-outs at the Medical Stores Department.

Procurement procedures varied between districts and from one health facility to another. All districts had prequalified private suppliers but the process and criteria for prequalification varied. After a publicly announced call for participation, a selection of prequalified suppliers is made, implying that they are listed as possible suppliers when need arises. These are the ones invited to give quotations for the products needed. That effectively ensures that participation is limited to the pool of prequalified suppliers for the duration of one year until the next prequalification tender is out calling for a new round of participation. However, the current observations revealed that some districts simply updated the list of suppliers annually without advertising while others advertised for prequalification. A comprehensive list of 77 prequalified suppliers for the whole Dar es Salaam region was recorded for the 2016/2017 financial year. Table 1 summarises the number of prequalified suppliers for each district in the Dar es Salaam region.

**Table 1:** Prequalified suppliers and their supply domain

District	No. of prequalified suppliers for medicines	No. of prequalified suppliers for laboratory reagents and equipment	No. of prequalified suppliers for medical equipment	Total^a^
Temeke	18	0	31	38
Kigamboni				
Kinondoni	16	19	21	29
Ubungo				
Ilala	7	7	7	10

^a^Some suppliers provide more than one supply domain (e.g. both medicines and medical equipment), hence the total lists the number of different suppliers

The number of prequalified suppliers ranged from 10 to 38 per district, but only 17 prequalified suppliers were utilised in the whole of the reviewed period. The criteria for choosing which supplier to invite to tender was unclear. The pool for prequalified suppliers consisted of large, known wholesalers/distributors; however, the majority of bids were from small suppliers. In addition, 33 non-prequalified suppliers were also given an invitation to tender and supply health commodities. The study found that a number of prequalified suppliers used for the supply of health commodities were not licensed by the authorities to do this type of business. Moreover, tenders were given to prequalified suppliers despite not always being prequalified to supply in a specific field. For example, it was observed that a supplier prequalified to supply laboratory equipment and reagents only was given an invitation to tender for the supply of medicines to compete with bidders who were prequalified for the supply of medicines. Furthermore, tenders were given to suppliers neither licensed nor prequalified to supply health commodities and some invoices revealed that suppliers charged Value Added Tax for healthcare supplies despite the government exempting such taxes. One supplier was licensed to supply health commodities, three were licensed to supply medicines and medical supplies and six were licensed to supply laboratory equipment, reagents and medical supplies. All ten suppliers were licensed health commodity wholesalers or retailers and six of them were also importers.

### Total funds used for the procurement of health commodities

The source of funds that public health facilities in Dar es Salaam used for the procurement of essential health commodities from private suppliers, in the review period, was derived from the Health Basket Funds, out-of-pocket payments and various health insurances, including the National Health Insurance Fund. Completed public health facility orders to private suppliers using complementary funds during the 2016/2017 financial year amounted to USD 663,491 (data obtained from facilities referred to in Table 2). Table 2 illustrates the total funds used with the number and type of sources indicated for each district. Most complementary funds were used by Ilala, Ubungo, Kinondoni and Temeke, in decreasing order.

**Table 2:** Funds used by each district for the indicated source type and numbers

District	No. of Sources	Source Type	Complementary funds used for the procurement of health commodities from private sources (USD)
Ilala Municipal	4	1 Referral Hospital 1 Primary Care Hospital 2 Health Centres	205,309
Kinondoni Municipal	3	1 Referral Hospital 2 Health Centres	133,251
Ubungo Municipal	3	1 Primary Care Hospital 2 Health Centres	189,196
Temeke	1	1 Referral Hospital	128,837
Kigamboni	2	1 Primary Care Hospital 1 Health Centre	6898
**Total**			**663,491**

### Private supplier assessment

The total number of orders, i.e., 72 to the 17 prequalified suppliers in the period between July 2016 and June 2017 were analysed. The order fulfilment rate by those private suppliers was 100% (Table 3).

**Table 3:** Summary of orders delivered by private suppliers to public health facilities in Dar es Salaam

Council	Suppliers	No. of orders	Fulfilment rate %
Temeke	3	21	100
Ilala	4	27	100
Kinondoni	4	6	100
Ubungo	4	12	100
Kigamboni	2	6	100
**Total**	**17**	**72**	**100**

The order fulfilment rate by the Medical Stores Department, as sourced from the electronic Logistics Management Information System (eLMIS) covering the reviewed period was as follows: approximately 34% of health facilities had a fill rate (had received their commodities) of over 75%, another 34% of health facilities had a fill rate of 50–75% and approximately 32% of the remaining health facilities had a fill rate of less than 50%.

A price comparison was made for a selection of most frequently ordered items from three of the prequalified suppliers as indicated demonstrating a strong price variation for some items such as Benzyl penicillin injections that deviated between − 22 to +17% from the average price of USD 16.5 (Table 4).

**Table 4:** Price comparison of selected popular items between three private suppliers during the 2016/2017 financial year

Description of item	Unit measure	Supplier 1 Price (USD)	Supplier 2 Price (USD)	Supplier 3 Price (USD)
Benzyl penicillin injection	*50 vials per pack*	19.4	17.3	12.9
Paracetamol 500 mg	*1000 tablets per pack*	5.2	6.5	5.2
Syringes 5 cc	*100P*	3.3	3.3	3.3
Malaria Rapid Diagnostic Test	*25P*	9.4	8.6	8.6

### Assessment of suppliers visited

A requirement for the suppliers to become prequalified and then shortlisted by the councils was that they employed the necessary healthcare professionals. These included pharmacists, pharmaceutical technicians, pharmaceutical assistants, laboratory technicians, biomedical technicians, laboratory assistants, assistant medical officers and clinical officers. Each of the 10 suppliers visited fulfilled this criterion. Moreover, the availability of health commodities that are commonly requested by health facilities and frequently out of stock at the Medical Stores Department was recorded at each of the 10 suppliers visited and ranged between 65.2 and 100%. For quality assurance purposes, the availability and use of standard operating procedures (SOPs) was assessed as well as the storage conditions by observations based on standard indicators of Good Storage Practice (GSP). Only one of the 10 suppliers visited had SOPs that governed routine activities within their warehouses, while the storage conditions of the 10 suppliers visited had met on average 91.7% of GSP criteria with a range of between 83.3 and 100%. The lead-times for medical supplies for the visited suppliers ranged between 1 and 45 days (as for all 50 suppliers during the period assessed). Their lead times for medicines, however, ranged between 1 and 14 days.

The customer base of the 10 suppliers visited, included government health facilities, non-governmental organisations, faith based health facilities, private health facilities and private pharmacies. Deliveries within Dar es Salaam were by means of company vehicles, motorcycles and hired vehicles and were not charged any additional transportation costs. For deliveries outside Dar es Salaam, a transport agency would be commonly deployed by the supplier and the cost shared between the supplier and customer. The suppliers accepted a variety of modes of payment that included cheques, Tanzania interbank settlement system (TISS), cash and direct deposits to the firm’s bank account. After sales service of medical equipment included installation, training and monitoring. The annual turnover of health commodities for the suppliers that were visited ranged from USD 43,122 to USD 8,624,407.

## Discussion

The presented work describes a situation analysis of the procedures and practices of procuring medicines and medical supplies from private suppliers in the Dar es Salaam region when those commodities are not available at the national Medical Stores Department. This assessment is the first step of the 12-step implementation sequence of the Prime Vendor System, successfully piloted and implemented in of Dodoma, Morogoro and Shinyanga regions. It provides arguments for a necessary system change in the procurement practice from private suppliers. The study results offers the rationale for regional authorities to identify one reputable private sector pharmaceutical supplier, through a transparent prequalification and procurement process, to engage as prime vendor to create a public–private partnership based on good procurement practice. Public healthcare facility orders not fulfilled by the Medical Stores Department could then be pooled at council level and purchased from the prime vendor at competitive rates. The regional prime vendors, in turn, agree to supply quality assured materials at comparable prices to the Medical Stores Department with good lead times.

The generous selection of 77 prequalified suppliers for the whole of Dar es Salaam has the potential to provide ample competition and therefore lower prices for medicines to be obtained from private suppliers. However, given the lack of transparency on the invitation to tender process, the value for money element could not be verified. Furthermore, the fact that the majority of the 17 prequalified suppliers used during the review period were small businesses, some of which not licensed to supply health commodities and 33 suppliers not even prequalified, renders the quality of the medical supplies and any medical or economic gains questionable [15]. The budget of Dar es Salaam is approximately 1.5 billion Tanzanian shillings (~USD 700,000), for the procurement of health commodities from private suppliers from the five sample districts, when Medical Stores Department stock-outs occur. This is approximately three times the budget that the regions of Dodoma, Morogoro and Shinyanga each have for the same. Considering this significant sum, it is concerning that legislation and regulations, for this type of procurement, are not strictly adhered to and enforced. However, with a lack of guidance and SOPs, in addition to lengthy bureaucratic processes, obtaining medical necessities via speedier alternative means appears to be considered the norm; it being a more practical and attractive option to many healthcare providers, even if it is illicit. Unfortunately, healthcare regulatory systems, especially those that are weak and/or poorly enforced are attractive targets for corruption, which have the potential to cause direct and serious harm to individuals and to society [16, 17]. In 2017, global expenditure on health reached USD 7.8 trillion and it is estimated that 10–25% of health expenditure is lost to corruption [16, 18, 19]. Strangely, according to the World Health Organization, it is uncommon for many countries to designate more than 0.1% of their health budgets to auditing and investigating corruption despite the considerable sums that could potentially be spared if they did [15]. Moderately high corruption levels are not unique to Tanzania; however, in 2019, Transparency International’s Corruption Perceptions Index ranked Tanzania 96th from 198 countries with a score of 37 out of 100, 0 being very corrupt and 100 being very clean [20]. The benefits of UHC will be lost if the general perception of public healthcare is poor, the medicinal quality questionable, corruption endemic and health outcomes compromised [21]. Poor quality of healthcare services, in particular, can discourage people to use them, even if they are financially covered, or they can lead to adverse health outcomes, both of which defy the point of UHC. High-quality health services, on the other hand, often lead to improved health outcomes, a positive user experience and more confidence in the system, therefore, positively re-enforcing a cycle of public support and sustained government financing. Furthermore, participation in community health insurance schemes, for example, is much more acceptable if the quality of services is high. This in turn helps sustain government financing and could help to lower the national incidence of catastrophic spending [21–24].

The main challenges to the procurement process from private suppliers observed in Dar es Salaam, i.e., bureaucracy and a lack of transparency and standardization, was observed in all Jazia PVS pilot regions prior to the implementation of the prime vendor scheme (i.e. Dodoma, Morogoro and Shinyanga) [10] (unpublished data). For Dodoma, at least, it has been shown that the prime vendor system has managed to simplify, shorten and standardize procurement procedures and render governance more transparent, while enhancing procurement capacity at all levels of the health system [10].

### Study limitations

This baseline study was not intended to be an in-depth investigation of health commodity suppliers. Instead, it was a situation assessment of the operational environment and conditions when purchases by public health facilities or districts from a private sector occurred. Some highly relevant qualitative documents could not be accessed and/or were not made available in some districts. Consequently, the information obtained within this assessment was mainly of descriptive and qualitative nature, based on comments, interviews, observations and perceptions. We can therefore not exclude biases and inaccuracies introduced by both public and private sector interviewees leading to difficulties in assessing lead times, for instance.

## Conclusion

This situation analysis of public–private procurement practices for medical commodities in Dar es Salaam outside Medical Stores Department provides valid arguments for a necessary system change in the procurement procedures from private suppliers. The study results offers the rationale for regional authorities to create a public–private partnership based on good procurement practice by identifying one reputable private sector pharmaceutical supplier to engage as prime vendor. The currently fragmented, cumbersome and poorly regulated procurement practice for health supplies from the private sector in Dar es Salaam when the Medical Stores Department is unable to supply needed commodities calls for the introduction of a more efficient supply system. Introducing the PVS in Dar es Salaam, anchored in existing government structures and successfully implemented in other regions, promises an efficient, transparent complementarity to the Medical Stores Department.

Simplifying and increasing the transparency and efficiency of procurement procedures from the private sector with a prime vendor system would not only help to better manage Medical Stores Department stock-outs but also help improve health care services overall, optimizing the use of limited resources, restoring confidence in the Tanzanian health care system and its path to achieving UHC and attaining its 2025 Development Vision.

## Data Availability

The datasets used and/or analyzed during the current study are available and can be accessed from the corresponding author on reasonable request.

## Abbreviations

UHC: Universal health coverage
HPSS: Health promotion and system strengthening project
Jazia PVS: Jazia prime vendor system
LPOs: Local purchase orders
eLMIS: electronic logistics management information system
SOPs: Standard operating procedures
GSP: Good storage practice
TISS: Tanzania interbank settlement system.
